# Pure Yolk Sac Testicular Tumour in an Adult: A Diagnostic Dilemma

**DOI:** 10.7759/cureus.74332

**Published:** 2024-11-24

**Authors:** Thomas R Fonseka, Charles Adewole, Teresa Mele, Anuj Goyal, Wai Gin Lee

**Affiliations:** 1 Urology, Royal Free Hospital, London, GBR; 2 Pathology, Royal Free Hospital, London, GBR; 3 Oncology, St Bartholomew's Hospital, London, GBR; 4 Andrology, University College London, London, GBR

**Keywords:** caring for people with learning difficulties, large scrotal mass, metastatic testicular cancer, pure yolk sac testicular tumour, testicular cancer with vascular complications

## Abstract

A rare case of a pure yolk sac testicular tumour presenting in an adult with learning difficulties is presented. Pure yolk sac tumours are much more common in children, but when they do occur in adults, onset can be both insidious and aggressive. The best practice for identification involves the precise use of ultrasound, blood tests for tumour markers and FDG-PET/CT imaging for staging. Dual-phase treatment in the form of radical inguinal orchidectomy performed expediently followed by chemotherapy is the mainstay of treatment in advanced cases.

## Introduction

Worldwide, the rates of testicular cancer are increasing; tumour subtypes now considered rare may well be seen more commonly in the future [1]. Testicular germ cell neoplasms are the most common cancer in young adult males. Testicular tumours can be divided into seminomas (the commonest type) and nonseminomas (including yolk sac tumours) [2]. Whilst yolk sac testicular tumours (YSTTs) are one of the most likely tumours to be encountered in children (accounting for approximately 70-80% of testicular malignancies), they are exceptionally rare in adults [3]. In adults, they can present at an advanced stage, mimicking the signs of an infective process [4]. If the tumour is misdiagnosed, then it may lead to a scrotal approach to surgery rather than an inguinal approach. The former risks upstaging the tumour as it could cause seeding. Awareness of this rare tumour subtype is therefore important to ensure the correct management is instigated in a timely manner.

The case of an adult male with a background of learning difficulties (LD) presenting with an advanced pure YSTT masquerading as Fournier’s gangrene is presented. The patient was originally taken to the operating theatre for a scrotal debridement to treat a presumed diagnosis of Fournier’s gangrene. However, instead of intra-scrotal pus, a complex scrotal mass with areas of necrosis was encountered. Intra-operative scrotal mass samples were sent to histopathology, which revealed a pure YSTT.

Following this challenging case, a review of the literature was conducted to identify cases where similar presentations had been encountered, including the management of men with both learning difficulties and testicular malignancy. We highlighted the best practices regarding both surgical and oncological management of this rare, but clinically significant, tumour type to help guide clinicians in the future when faced with a similar diagnostic dilemma. 

## Case presentation

Presentation and initial management

A 53-year-old man with a background of Asperger’s syndrome had been previously seen in the andrology clinic with a six-year history of left scrotal swelling. He had no other documented comorbidities and lived alone, being of performance status 0. A provisional diagnosis of a hydrocoele was made and he was listed for surgical excision pending an ultrasound scan of the scrotum.

Before the ultrasound was performed, he presented to the emergency department with general malaise. On examination, the left hemiscrotum was markedly swollen to a diameter of approximately 12cm and was firm on palpation. He was afebrile but blood tests showed an elevated CRP (74mg/L) and he was commenced on intravenous gentamicin for a likely scrotal abscess.

Imaging

**Figure 1:**
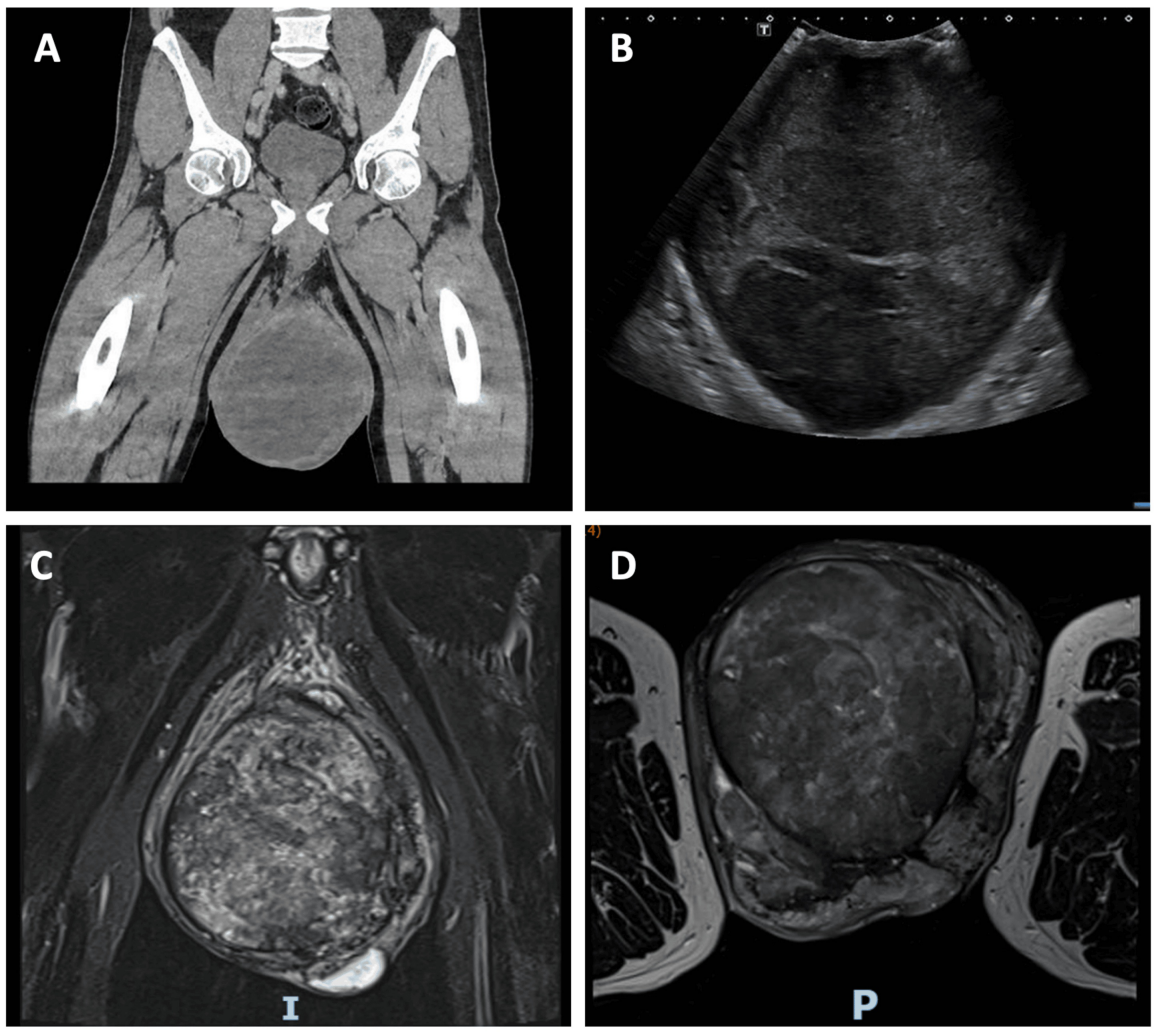
Summary of the three imaging modalities used A: coronal view contrast-enhanced CT; B: greyscale view ultrasound; C: coronal view T2-weighted MRI; D: axial view T1-weighted MRI

As the patient presented to the emergency department out of hours, it was not possible to perform an inpatient ultrasound. Therefore, an emergency CT scan was requested which was performed during admission. Contrast-enhanced CT revealed bilateral scrotal swelling with small volume reactive groin lymph nodes and no radiological evidence of necrotising fasciitis (Figure 1A).

On Day 1 of the hospital admission, an area of skin on the most dependent part of the scrotum had turned brown in colour. The patient remained afebrile and an ultrasound scan was performed to obtain more information on the underlying scrotal pathology (Figure 1B). The scan revealed signs of a complex scrotal abscess. It was thought less likely to represent a mass given the lack of Doppler signal. The testes could not be identified separately. By the time of the scan, the skin changes had progressed and he was promptly taken to theatre for debridement on the suspicion of Fournier’s gangrene. 

Surgical management

**Figure 2:**
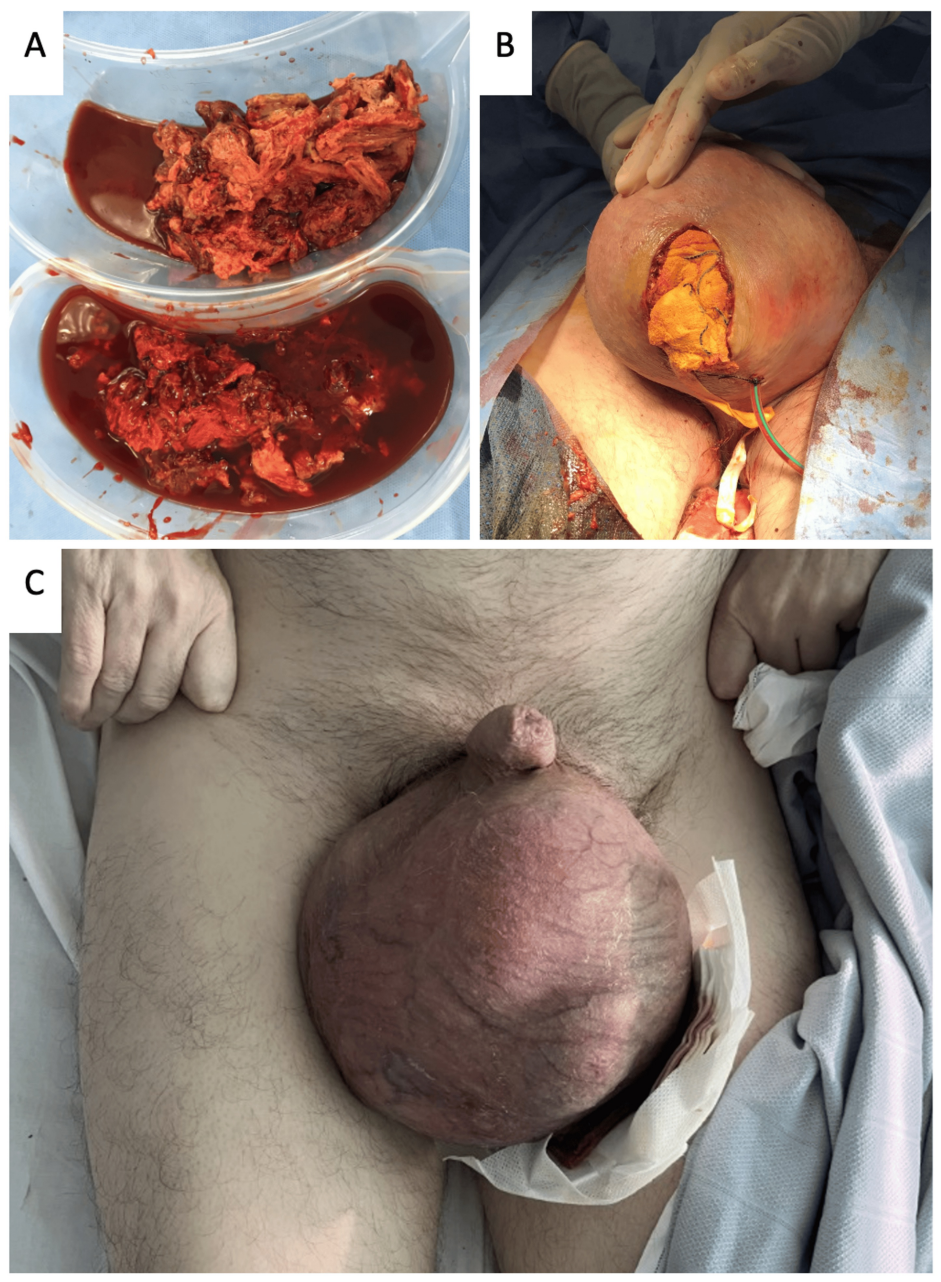
Peri-operative images A: Intra-operative images of the tissue removed from the scrotum B: Packing of the scrotum and drain placement after debridement C: Post-operative image of the swollen scrotum

During the operation, a complex mass with areas of necrosis was found within the scrotum and concern was raised regarding the possibility of malignancy (Figure 2A). The decision was therefore made to sample the intra-scrotal tissue for diagnostic purposes. The scrotum was then packed and a drain was placed (Figure 2B). Post-operative appearances of the scrotum can be seen in Figure 2C.

Whilst awaiting results from the tissue samples, an MRI scan of the scrotum was performed in an attempt to ascertain whether this was indeed a pyocoele or a malignant lesion. The scan did not confirm malignancy but suggested that the lesion had a cystic morphology with probable haemorrhage throughout (Figure 1C-1D). Staging CT showed no metastases. No single scan was able to elucidate the left testicle. 

Histopathology

**Figure 3:**
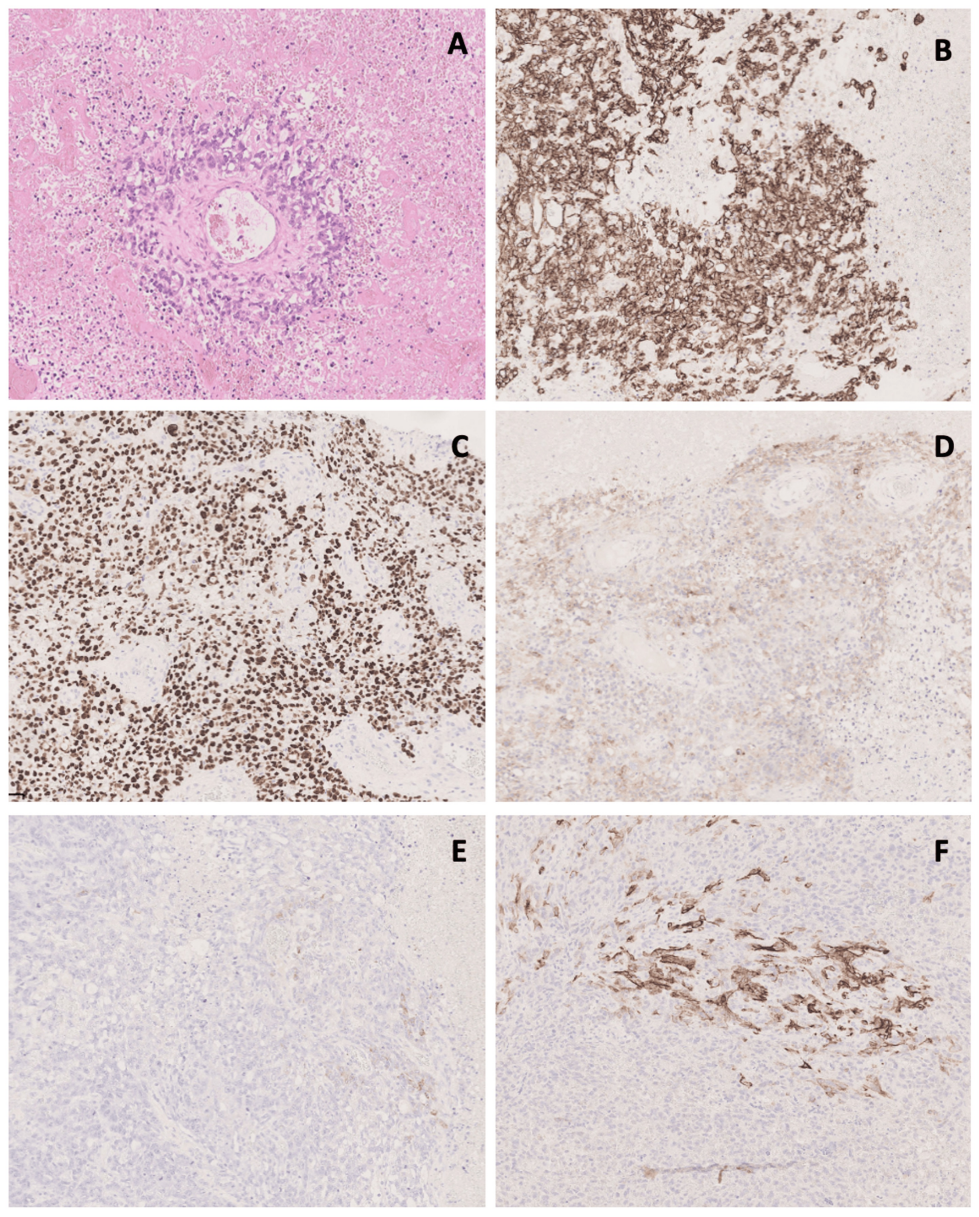
Photomicrograph of the tumour Hematoxylin-and-eosin photomicographs (400X) show the pathognomonic Schiller-Duval body (A) on a background of sheets of pleomorphic cells with extensive necrosis. Immunohistochemistry (400X) shows tumour cells reactive to pancytokeratin (MNF116) (B), SALL4 (C) and Glypican-3 (D). There was very focal AFP positivity (E) and focal CK7 positivity (F).

The intra-scrotal tissue showed an extensively necrotic neoplasm composed of sheets of pleomorphic cells lacking glandular, squamous, and neuroendocrine differentiation (Figure 3). There was brisk mitotic activity and widespread apoptosis. Viable tumour cells surrounded the small blood vessels, representing Schiller-Duval bodies, which are pathognomonic for yolk sac tumours. In other areas, the cells had a more spindled and sarcomatoid appearance. The scrotal skin showed ulcerated squamous epithelium with intense acute inflammation in the underlying stroma, with infiltration by a tumour similar to that in the intra-scrotal tissue.

Extensive sampling was done to exclude the presence of other germ cell components. This yielded no additional findings. The case was reviewed at the supra-regional level and the result was corroborated with the conclusion that this was a pure yolk sac tumour showing changes indicative of aggressive behaviour.

Further management

Tumour markers were sent post-operatively and showed raised lactate dehydrogenase (LDH) at 437 U/L, raised alpha-fetoprotein (AFP) at 6,964.0 kU/L and normal human chorionic gonadotropin (HCG) at 1.1 U/L. CT imaging of the chest, abdomen and pelvis showed no metastases. He was referred to a supra-regional cancer management centre where he received three cycles of baby-BOP (low-dose bleomycin, vincristine, and cisplatin) with significant tumour marker response (AFP 2081 and normal LDH two weeks after commencing treatment). This low-dose regimen was preferred over conventional BEP (bleomycin, etoposide, cisplatin) to minimise toxicity, in particular, the risk of infection and bleeding as at the time of treatment commencement the scrotal mass was deeply ulcerated. Unfortunately, he subsequently died approximately six weeks after initial presentation with the post-mortem revealing the cause of death as an acute myocardial infarction. This was likely related to a multifactorial combination of undiagnosed coronary artery disease, large tumour burden and the use of platinum-based chemotherapy.

## Discussion

Findings from the literature regardinginitial presentation, diagnosis and management

The literature was reviewed to identify any pertinent papers on the diagnosis and management of pure YSTT in adults. Table 1 summarises all 13 articles identified in the review which reported on a total of 27 cases.

**Table 1:** Patient demographics, diagnostic information, treatment modality and peri-operative outcomes Abbreviations: BEP = bleomycin, etoposide and cisplatin, CT = computed tomography, FDG-PET = fluorodeoxyglucose -positron emission tomography, RIO = radical inguinal orchidectomy, RPLND = retroperitoneal lymph node dissection, USS = ultrasound scan

Article	Type	Country	No. of cases	Age (years)	Imaging modality	Time to diagnosis (months)	Tumour size (cm)/ description	Treatment	Disease-free survival (months)
Munver et al., 2000 [5]	Case report	USA	1	41	CT	96	‘Enormous’	RIO + reduction scrotoplasty + 4 cycles BEP	24
Foster et al., 2000 [6]	Case series	USA	12	Mean = 29, Range = 14-41	USS, CT	N/A	N/A	RIO + RPLND (12) + 2-3 cycles BEP (5)	Stage 1 tumours = 60, Stage 2 tumours = 59
Ucer et al., 2016 [7]	Case report	Turkey	1	35	USS, CT, PET CT	1	10	RIO + chemotherapy	3
Khan et al., 2014 [8]	Case report	India	1	33	USS	N/A	7	RIO	N/A
Janugade et al., 2019 [9]	Case report	India	1	46	CT	1	11.2	Laparotomy + surgical excision. Patient died before chemotherapy	1
Asci et al., 2022 [4]	Case report	Turkey	1	35	CT	3	N/A	Surgical debridement + chemotherapy	6
Patel et al., 2023 [10]	Case series	USA	4	Mean = 36, Range = 25-68	USS (n=4) CT (n=1), MRI (n=1)	Case 1 = 2 months, Case 2 = N/A, Case 3 = 2 months, Case 4 = N/A	Case 1 = 5.8cm, Case 2 = 16.8cm, Case 3 = 5.2cm, Case 4 = ‘large’	RIO (4) RIO + chemotherapy (3)	Case 1=12, Case 2 = 12, Case 3= not reported, Case 4 = 3 months
Garcia-Gomez et al. 2021 [11]	Case report	Spain	1	35	USS, CT, FDG-PET	N/A	N/A	RIO + BEP	N/A
Medica et al., 2000 [12]	Case report	Italy	1	44	USS, CT	N/A	6.5	RIO + RPLND	6
Behera et al., 2019 [13]	Case report	India	1	37	USS, CT	84	N/A	RIO + 4 cycles BEP	48
Wang et al., 2015 [14]	Case report	China	1	30	USS, CT, FDG-PET	84	N/A	RIO, patient declined further treatment with BEP	N/A
Nia et al., 2021 [15]	Case report	USA	1	32	N/A	N/A	N/A	RIO + RPLND + BEP at primary presentation aged 15 years.	204
Benjelloun et al., 2022 [16]	Case report	Morocco	1	37	USS, CT	2 months	N/A	RIO	6

The mean age at diagnosis was 36.8 years (range = 14-68 years). Six cases presented at an advanced stage with a mean time to diagnosis of 34.1 months (SD = 43.2 months) [4-8,15]. In just over a quarter of cases, an initial diagnosis other than neoplasm was made (Table 2) [4,5,7,8,11-13]. Of these cases, 43% were diagnosed with epididymo-orchitis [5,7,13], 29% with scrotal abscess [11,12], 14% with testicular trauma [8] and another 14% with Fournier’s gangrene [4]. 

**Table 2:** Initial diagnosis at presentation for all cases not diagnosed as neoplasm (n=7)

Top differential diagnosis	Percentage
Epididymo-orchitis	43%
Scrotal abscess	29%
Testicular trauma	14%
Fournier’s gangrene	14%

The average tumour diameter was 8.92 cm (SD = 4.11cm) for all reported cases. There was insufficient data to assess whether greater tumour diameter positively correlated with a more advanced stage at diagnosis. 

Imaging modalities included ultrasound as the first line followed by CT and positron emission tomography (PET) CT for staging and surveillance. All cases received radical inguinal orchidectomy with or without adjuvant BEP (bleomycin, etoposide, cisplatin) chemotherapy. There was one case of wound infection and sepsis following surgery which resulted in patient death [9]. There were no other significant (Clavien-Dindo classification grade ≥ 2) complications from surgery or other complications from chemotherapy reported.

Diagnostic Challenges

Most cases of YSTT are seen in children under the age of three years; in adults, the yolk sac element is usually mixed with other germ cell components. Pure YSTTs are exceptionally rare and have an incidence of 2.4% in adults presenting with a testicular lesion [9]. There are no specific risk factors for yolk sac tumours other than the general risks for testicular tumours (undescended testes, family history, aged between 20 to 35, caucasian ethnicity and HIV infection). Indeed, cryptorchidism was present in two of the 27 cases [9,14].

Clinical presentation is often only of a testicular mass without any other specific symptoms. Pure YSTTs are considered an aggressive tumour with a high risk of metastases and therefore multi-modality treatment is often required [10]. Foster et al. report in a series of 12 cases that the adult tumour is far more aggressive than the juvenile form, with one-third of cases being stage 2 at presentation [6,16]. Recurrence after primary treatment can be fatal and therefore full multi-modal treatment should be advocated when appropriate [15]. Nia et al. report a 32-year-old male presenting with brain metastases 17 years after initial multi-modal treatment for a YSTT. Despite craniotomy and tumour resection, the patient died after approximately two months.

Several studies reported a time to diagnosis of many years from the initial presentation [5,13,14]. Where the patient is not originally diagnosed with a testicular neoplasm, the diagnosis tends to be of an infective process with epididymo-orchitis found to be the most common diagnosis. The average tumour diameter of 8.92cm (SD = 4.11cm) across all studies was much larger than usual. Song et al. analysed 247 men with malignant testicular tumours and reported an average tumour diameter of 5.4mm ± (standard deviation = 0.2mm) [17]. The same group also reported larger tumour diameter as a positive predictor for malignant tumours in all men with testicular tumours. The large tumour diameter at presentation in the present review is in keeping with the aggressive nature of the disease. Clinicians should therefore be wary of men presenting with large scrotal masses, ensuring to rule out signs of malignancy.

Imaging provides invaluable insight into both the underlying pathological process as well as the signs of metastases. Whilst colour Doppler ultrasound scan (CDUS) is the current first-line choice of imaging for the acute scrotum, fluorodeoxyglucose (FDG)-PET CT can aid in formulating a diagnosis and also provides information on prognosis by identifying distant metastases [11]. In the present case, imaging never elucidated the testicle and it appears that it was completely replaced by necrotic, haemorrhagic tissue, as has been reported by Khan et al [8]. This was also described by Wang et al. where an ultrasound scan of a YST indicated poor blood flow [14]. This is contrary to ultrasonographic features in children where increased vascularity is often found. In adults, it is important to adjust the scale on Doppler ultrasound machines to give the best possible chance of detecting blood flow and hence identifying a malignant process. While the blood flow is often diminished in the late stages of the tumour, detecting some flow may be pivotal in lending more weight to a malignancy rather than an infective process [14]. 

Management Outcomes Reported in the Literature

One case reported a fatal recurrence 17 years after being treated for a primary pure YSTT aged 15 years [15]. The time period of follow-up was variable but the average minimum disease-free survival across all reporting studies was 27.3 months (SD = 57.2 months) [4-7,9,10,12,13,15]. Foster et al. reported YSTTs in 12 patients and found a cure rate of 87.5% for stage 1 tumours and 66% for stage 2 tumours [6]. One study reported that a patient died from sepsis following a wound infection one month after diagnosis [9]. Another study reported a patient death from metastatic disease three months after diagnosis, implying a poorer prognosis compared to other germ cell tumours [10].

Testicular Cancer in Men With Learning Difficulties

As in the present case, symptoms can be of a slow, insidious onset leading to patients presenting late with extensive necrosis and haemorrhage of the tumour [8]. The background of Asperger's syndrome and LD is likely to have been a factor in causing the late presentation. LD patients were found to have a poorer prognosis, with 10-year testicular cancer-specific survival of 88.4% (95% confidence interval 84.5-92.4%) in the LD group versus 96.8% (95% confidence interval 96.6-97.1%) in the non-LD group [18]. The poorer prognosis was largely attributed to a lack of self-examination and late presentation.

Testicular self-examination poses unique challenges to those with LD. Using an individualised approach to overcoming barriers to health promotion is paramount in ensuring testicular tumours are detected in a timely manner in this population. A multidisciplinary team approach to addressing issues around consent to intimate examination and existing legislative frameworks must be utilised to ensure these patients receive equitable healthcare. Special consideration must be taken for conducting a testicular examination in those with LD as this intimate examination may be especially distressing in this population. Adjusting the clinical environment, using pictures or videos, utilising examination dolls, providing easy-to-read written information and utilising the assistance of learning difficulty nurses are all ways to make this examination less daunting for this patient population. 

Best Practice Surgical Management of Advanced YSTT

Differentiating between scrotal abscess and malignancy is essential in deciding whether a scrotal or inguinal approach is required. The former approach risks tumour seeding and up-staging of a cancer if a malignancy is present but the distinction can be challenging. For acute scrotal pathology, every effort must be made to ensure the correct diagnosis is made before embarking on surgical intervention.

In the present case, prompt surgical intervention was necessary due to signs of tissue necrosis and impending Fournier’s gangrene. Asci et al. also reported an advanced YSTT presenting as Fournier’s gangrene which necessitated surgical debridement [4]. The scrotum, in these late stages, can be markedly increased in size [5]. The authors hypothesise that this may lead to a pressure necrosis of the dependent skin of the scrotum, mimicking the signs of Fournier’s gangrene. In the case reported, while the skin of the scrotum appeared blackened, the patient remained afebrile and inflammatory markers were not markedly elevated. Therefore, it was not a classical presentation of Fournier’s gangrene. 

Best Practice Oncological Management of Advanced YST

Oncological management of metastatic YSTT follows the general principles of management of metastatic non-seminomatous germ cell tumours. Risk classification and prognostic group are based on the International Germ Cell Cancer Collaborative Group (IGCCCG) prognostic classification and its recent updates. The risk group at diagnosis is based on the level of tumour markers, location of the primary tumour (testicular vs non-gonadal) and site of metastatic disease (absence vs presence of non-pulmonary visceral metastases) [19].

The IGCCCG updated model on NSGCT reports an estimated five-year progression-free survival of 90% in the good prognostic group, 78% in the intermediate and 54% in the poor prognostic group with respective five-year overall survival of 96%, 89% and 67% [19].

Standard treatment in advanced disease is based on three cycles of BEP chemotherapy (or four cycles of EP) in the good prognosis group vs four cycles of BEP in the intermediate or poor risk group. Low-dose induction chemotherapy (baby-BOP) can also be used in patients with extensive metastatic disease who are symptomatic at presentation with poor performance status. The low-dose regimen can avoid significant neutropenia, thus lowering the risk of infection in an already unwell patient. 

## Conclusions

This case demonstrates the challenges of diagnosing a pure YSTT in adults. Testicular cancer in patients with LD is associated with poorer outcomes and increased mortality. The case reported was particularly challenging given the initial diagnostic uncertainty, which is not unusual for these tumours. Appropriate imaging is pivotal in the diagnosis and adjusting the scale on the Doppler ultrasound machine to pick up on very diminished blood flow within the scrotum is vital. Utilising FDG-PET CT to identify metastases can also be beneficial in planning treatment. As rates of testicular cancer continue to increase worldwide, this is an important disease entity that will likely become more common in future. A multi-modal treatment strategy is advocated to treat these aggressive tumours and optimise management.
